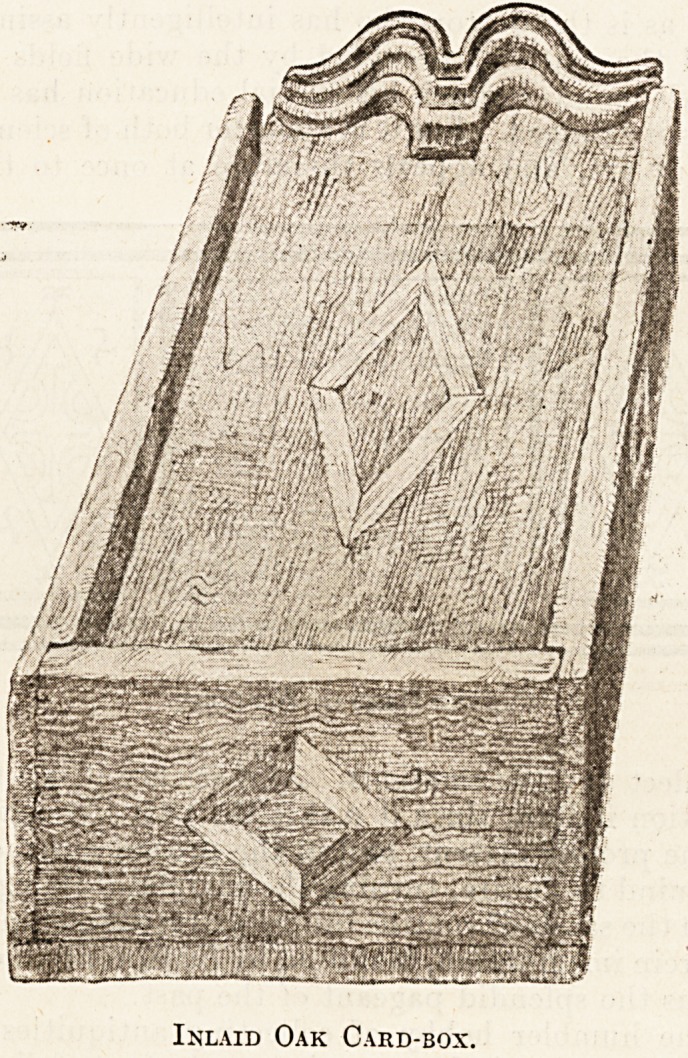# Archæology and the Collection of Antiques

**Published:** 1907-11-02

**Authors:** 


### The Practitioner's Relaxations and Hobbies.

### ARCHÆOLOGY AND THE COLLECTION OF ANTIQUES.

Archæology is perhaps of all forms of recreation the most suited to a man of science and culture, such as is the doctor who has intelligently assimilated the material provided by the wide fields of knowledge over which his special education has of necessity ranged. For it is a matter both of science and of art, and appeals therefore at once to the intellect and the emotions. As a science it is a recreation indeed, since it makes the past live again in the prosaic present, and in the process abstracts the mind from care, turning the current of thought from the sands of routine into more fertile channels wherein imagination, manured by facts, reproduces for us the splendid pageant of the past.

Carved Head of Bedstead.

The humbler hobby of collecting antiquities is a particularly suitable pursuit for a doctor to follow. The daily round will often introduce him to relics of the past, especially those of domestic art, as represented in ancient furniture and utensils, in antique objects of decoration, as glass paintings, samplers or other old needlework, and in old engravings, mezzotints, oil or water-colour paintings. Some of these are obtainable—at a price; others are clung to with an attachment which may be born of affection, but which is as often merely due to an exaggerated opinion of their value, engendered by paragraphs in local newspapers recording "finds" of valuable objects in surroundings where they had previously been looked upon as "unconsidered trifles." Certainly, it is often in quite unlikely places the doctor may come across some object of antiquity, art, or quaint beauty, or of all combined, and may be fortunate enough to secure some one or other of them at a reasonable price before a dealer comes upon the scene and snaps it up. During many years of collecting I have accumulated not a few articles of antiquity and beauty, and of use withal. Thus I was once called to visit a sick man in the attic of an unpretentious country public-house, which nevertheless bore the aspect of an old coaching inn. While taking his temperature I noticed that the head of his tumble-down wooden bed was formed of a piece of carved panelling apparently of Tudor date. That this was its original position is unlikely; but its origin was quite unknown. Of its three panels the central was on a lower plane than those on either side, and was movable in one direction behind, sliding in grooves visible at the back. Who can tell what secrets this old sliding panel once hid? The carving is of the incised variety, the most perfect of its kind I have ever seen, betokening a strong, sure hand, a keen eye, and tools of the sharpest edge. For incised carving—as I can say from experience—is very difficult of execution; much more so than the raised kind, which is largely a matter of patience. I was allowed to become the possessor of this old woodwork on offering hard cash; not perhaps too cheaply, since I never received any payment for several visits paid to the sick wayfarer.

There is but limited and quite chance opportunity for the collector who lives in the home counties to pick up what may be called bargains. For the dealers in the towns, even quite small ones, are very active; and the very auctioneers' porters act on their standing commissions, at the remotest farmhouse sales, to buy up such things as mezzotints, regardless of condition; the very high prices obtainable for good ones more than counterbalancing losses on those in poor, or even damaged, state. Nevertheless a doctor may occasionally pick up some object of antiquity of more than usual interest or beauty, in spite of dealers, of other collectors, or of the ravages of time. Old oak chests are perhaps the articles of ancient furniture most likely to be met with. But it is rarely that one is so fortunate as I was when I became the purchaser of the very beautiful one here illustrated.

A Fine Old Oak Chest.

So little did its late owners value it that they used it to keep potatoes in, and it was in daily danger of damage from the banging up against it of great milk cans. By the offer of a new, larger, and stronger chest, together with a certain pecuniary addition, I obtained possession of it. It is much older than the usual old oak chests one meets with, the design in the centre of the panels being of an early character, and having a strong resemblance to a chest bearing a date of Henry VIII.s reign depicted in an old volume of that bygone periodical "The Mirror." Its warm brown colour is natural, the same within as without, and the sawdust of a portion that required repair was of the tint of ground coffee berries.

Doctors have also another—only too frequent—chance of acquiring objects of antiquity—namely, by taking them in discharge of otherwise hopeless debts. In this way I became the possessor of the small chest or coffer, ornamented with incised carving, as here illustrated.

Another and more ancient relic is a small box ornamented with coloured-wood inlay, acquired in the same way. It was described to me as a box to hold a prayer-book, and indeed such was the use to which its former possessor put it, but I am strongly inclined to think that its purpose was more profane—namely, to keep, as I do, a pack of cards in. By the by, cards are called by certain puritans "the Devil's Prayer-book." Whatever its original use or purpose, it is a quaintly pleasing little box, five inches long, four broad, and two deep, the sliding lid three-quarters of an inch longer, with a carved end of a double ogee outline. The centre of the lid of each end and each side has an inlaid

A Coffer with Incised Covering.

diamond pattern of two different coloured pieces of wood, an inch long and $\frac{1}{6}$ inch wide, light yellow and dark brown (almost black) pieces in each diamond arranged alternately on the warm brown of the oak, giving a very pleasing effect.

Such are a few of the articles of old oak which I have collected. There is, of course, a wider field open to the collector, in spite of the modern drawbacks to which I have referred; not only in other pieces of furniture of antique make, as tables, chairs, and bureaus, but also in pewter, copper, and brass candlesticks (of which I have nearly two dozen), brass fenders, old needlework, *et hoc genus omne*. Old silver does not, alas, often come into the possession of the poor practitioner, so he must needs be

Inlaid Oak Card-box.

content with plate or pewter, and some objects of ancient art of that nature are by no means to be despised. Old china, too, affords a most fascinating field for the collector's energies, presenting many relics of the past of great beauty and interest, and some value. But this is a subject too wide to be entered upon in this article.

W. H. Legge.

## Figures and Tables

**Figure f1:**
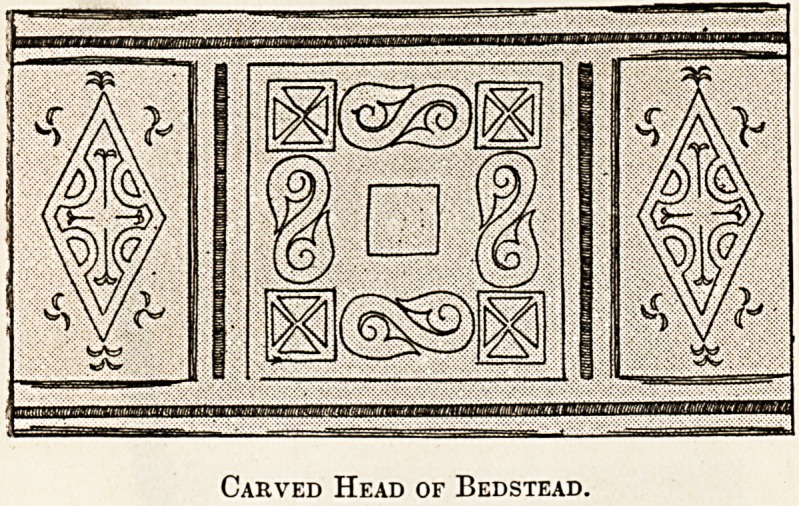


**Figure f2:**
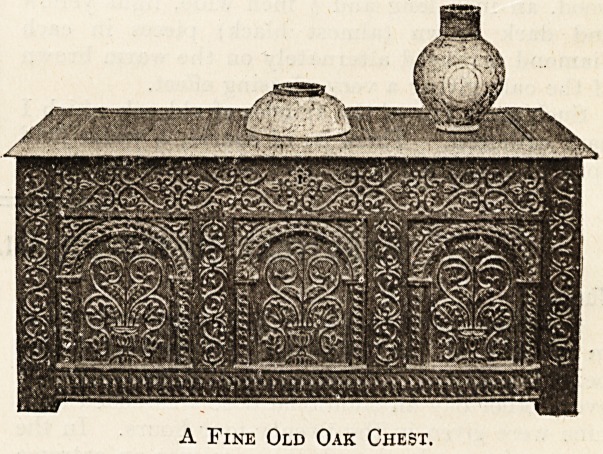


**Figure f3:**
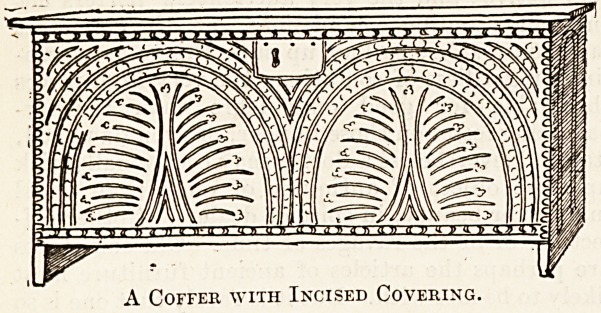


**Figure f4:**